# On Multilabel Classification Methods of Incompletely Labeled Biomedical Text Data

**DOI:** 10.1155/2014/781807

**Published:** 2014-01-23

**Authors:** Anton Kolesov, Dmitry Kamyshenkov, Maria Litovchenko, Elena Smekalova, Alexey Golovizin, Alex Zhavoronkov

**Affiliations:** ^1^Center for Pediatric Hematology, Oncology, and Immunology, Moscow 117997, Russia; ^2^Moscow Institute of Physics and Technology, Moscow 117303, Russia; ^3^The Biogerontology Research Foundation, Reading W1J 5NE, UK; ^4^Chemistry Department, Moscow State University, Moscow 119991, Russia

## Abstract

Multilabel classification is often hindered by incompletely labeled training datasets; for some items of such dataset (or even for all of them) some labels may be omitted. In this case, we cannot know if any item is labeled fully and correctly. When we train a classifier directly on incompletely labeled dataset, it performs ineffectively. To overcome the problem, we added an extra step, training set modification, before training a classifier. In this paper, we try two algorithms for training set modification: weighted k-nearest neighbor (WkNN) and soft supervised learning (SoftSL). Both of these approaches are based on similarity measurements between data vectors. We performed the experiments on AgingPortfolio (text dataset) and then rechecked on the Yeast (nontext genetic data). We tried SVM and RF classifiers for the original datasets and then for the modified ones. For each dataset, our experiments demonstrated that both classification algorithms performed considerably better when preceded by the training set modification step.

## 1. Background and Significance

Multilabel classification with supervised machine learning is a widespread problem in data analysis. However, very often, we have to perform multilabel classification when we are not guaranteed that our training set itself is perfectly preclassified. This is especially actual in the the case of national biomedical grants with ambiguous classification schemes. A particular grant may belong to several classes or may be miscategorized in the case of a keyword-based classification scheme.

An interesting illustration is the project titled “Levels of Literacy of Men with Prostate Cancer.” This project may be classified by an algorithm as “prostate cancer,” “cancer biomarkers,” or “cancer education” whereas a researcher would consider it appropriately in relation to literacy. This kind of context makes the generation of training sets more complicated and costly. Many experts need to collaborate extensively in the selection of the full set of document categories from the large number available for classification. Since such collaboration seldom happens, we end up assigning an incomplete set of categories to the training set document.

When a document that is relevant to a particular class *A* does not bear its label, it turns into a negative instance of class *A* during the learning process. As a consequence, the decision rules are distorted and the classification performance degrades.

With an increase in the amount of textual information in the biomedical sphere, such problems become recurrent and need our attention. For example, about half a million new records are added each year on PubMed and thousands of research initiatives funded by grants are conducted annually around the world. Grant application abstracts are usually made public and the IARP project adds over 250 thousand new projects each year.

In addition to classifying publication abstracts and grant databases, methods described in this paper may be applied to other classification tasks in biomedical sciences as well.

## 2. Objective

In this article, we address the problem of classification when for each training object some proper labels may be omitted. In order to understand the properties of incompletely labeled training set and its impact on learning outcomes, let us consider an artificial example.

**Figure 1:**
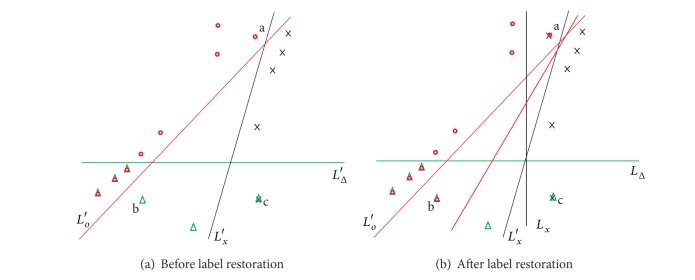
Decision rules before and after missing label restoration.

Figure 1(a) shows the initial training set for the multilabel classification task for 3 classes of points on the plane. In our work, we used Support Vector Machine (SVM) classification, which is a popular classical classification method with a broad range of applications ranging from text to tumor selection [1] gene expression classification [2] and mitotic cell modeling [3] problems.

With the Binary Relevance [4] approach based on a linear SVM, we can obtain the decision rules for the classes of crosses, circles, and triangles. Please note that this is an example of an error-free classification. Let us assume that object a really belongs to “crosses” and “circles” and object a belongs to “crosses” and “triangles”. But in real life the training set is often incompletely labeled. Figure 1(a) shows us such a situation, when object a is labeled only as “circle” (“cross” is missed) and object b is labeled only as “triangle” (missed “circle”).

In Figure 1(b), the missing tags and bold lines are added and the new decision rules for the classes of crosses and circles after recovering lost tags are depicted. This example shows that in the case of incompletely labeled dataset a decision rule may be quite distorted and have a negative effect on the classification performance.

In order to reduce the negative impact of incompletely labeled datasets, we proposed a special approach based on *training set modification* that reduces contradictions. After applying the algorithm to a real collection of data, the results of the Support Vector Machine (SVM) and Random Forest (RF) classification schemes improved. Here, RF is known to be one of the most effective machine learning techniques [5–7].

To address the incompleteness of training sets, in this paper we shall describe a new strategy for constructing classification algorithms. On the one hand, the performance of this strategy is evaluated using data collections from the AgingPortfolio resource available on the Web [8]. On the other hand, its effectiveness is confirmed by applying it to the Yeast dataset described below.

Several methods like data cleaning [9], outlier detection [10], reference object selection [11], and hybrid classification algorithms [12] for improving performance have been proposed for training set modification. To date, the ability of these approaches to provide real text classification has not been sufficiently studied. Furthermore, none of these methods of training set modification is suitable for solving classification problems with an incompletely labeled training set.

## 3. Methods

The already-proposed algorithms are based on the following three assumptions about totally new input classifier data.

1. A large number of training set objects are assumed to have an incomplete set of labels. By definition, a * complete set of labels* is a set which leads to a perfect consensus among experts regarding the impossibility of further adding or removing a label from a document in the data collection.
2. Experts are not expected to make an error in assigning category labels to documents. That is to say, the training set generation may involve errors of type 1 only (checking hypotheses of the type “object *d* belongs to category label cl”).
3. The compactness hypothesis is assumed to hold. This means similar objects are likely to belong to the same categories as compact subsets located in the object space. The solution of a classification problem under these assumptions requires that an algorithm treat document relevancy on the basis of data geometry.

We developed alternative approaches because the algorithms for training set modification were not designed to work with these assumptions. Then, we used the following two detailed algorithms in our experiments:

1. the method based on a recent soft supervised learning approach [13] (was labeled as “SoftSL”);
2. weighted k-nearest neighbour classifier algorithm (labeled as “WkNN” [14–16]).

These algorithms use the nearest neighbour set of a document which is in line with our third assumption.

The first step in the modification of the training set involves the generation of a set PC of document-category relevancy pairs overlooked by the experts:

(1) $$\begin{array}{c} \text{PC}=\left\{\left(d,\mathrm{cl}\right)\;|\;\psi \left(d,\mathrm{cl}\right)=1\right\}, \end{array}$$

where *d* is a document, cl⁡ is a class label (category), and *ψ* is the function of our training set modification algorithm (WkNN or SoftSL).

Consider

(2) ψ(d,cl⁡) ={1if  our  algorithm  places  document  d  into  class  cl⁡;0otherwise.

Then, two possible outcomes are considered:

1. complete inclusion of PC into the training set (option was denoted as “add”).
2. exclusion of document *d* from the negative examples of the category cl for all relevancy pairs (*d*, cl⁡) ∈ PC (option was denoted as “del”).

The modified training set will still contain the objects which, according to the algorithm *ψ*, do not belong to the set labeled by the expert. It is possible to find the next missing labels in the documents of the training set.

### 3.1. SoftSL Algorithm for Finding Missing Labels

In this section, we outline the application of a new graph algorithm for Soft-supervised learning, also called SoftSL [13]. Each document is represented by a vertex within a weighted undirected graph and our proposed framework minimizes the weighted Kullback-Leibler divergence between distributions that encode the class membership probabilities of each vertex.

The advantages of this graph algorithm include direct applicability to the multilabel categorization problem as well as improved performance compare to alternatives [14]. The main idea of the SoftSL algorithm is the following.

Let *D* = {*D*_*l*_, *D*_*u*_} be a set of labeled and unlabeled objects, with

(3) $$\begin{array}{c} D_{l}={\left\{\left(x_{i},y_{i}\right)\right\}}_{i=1}^{l};\;\;D_{u}={\left\{\left(x_{i}\right)\right\}}_{i=l+1}^{n}, \end{array}$$

where *x*_*i*_ is the input vector representing the objects to be categorized and *y*_*i*_ is the category label.

Let *G* = (*V*, *E*) be a weighted undirected graph. Here, *V* = 1,…, *n* where *n* is the cardinality of *D* and *E* = *V* × *V*, and *ω*_*ij*_ is the weight of the edge linking objects *i* and *j*.

The weight of the edge is defined as

(4) $$\begin{array}{c} w_{ij}=\{\begin{array}{cc} \text{sim}\left(x_{i},x_{j}\right), & \text{if}\;\;j\in K\left(i\right);\\ 0, & \text{if}\;\;j\notin K\left(i\right). \end{array} \end{array}$$

Here, sim(*x*_*i*_, *x*_*j*_) is the measure of similarity between *i*th and *j*th objects (e.g., cosine measure), and *K*(*i*) is the set of k nearest neighbours of object *x*_*i*_.

Each object is associated with a set of probabilities *p*_*i*_ = (*p*_*i*_^*t*^)_*t*=1_^*m*^ of belonging to each of the *m* classes *L* = {cl_*i*_}_*i*=1_^*m*^. According to information from *D*_*l*_, we determined the probabilities {*r*_*i*_ = (*r*_*i*_^*t*^)_*t*=1_^*m*^}_*i*=1_^*l*^ that documents {*x*_*i*_}_*i*=1_^*l*^ belong for each of m classes, thus *r*_*i*_^*t*^ > 0 if (*d*_*i*_, cl_*t*_) ∈ *D*_*l*_. Each labeled object also has a known set of probabilities *r*_*i*_, assigned by the experts. Our intention is to minimize the misalignment function *C*_1_(*p*) over sets of probabilities:

(5) C1(p)=∑i=1lDKL(ri,pi) +μ∑i=1n∑j∈K(i)ωijDKL(pi,pj)−ν∑i=1nH(pi),DKL(pi,pj)≝∑t=1mpitlog⁡pjt,H(pi)≝∑t=1mpitlog⁡pit,

where *D*_KL_(*p*_*i*_, *p*_*j*_) means Kullback-Leibler distance and *H*(*p*_*i*_) means entropy.

*μ* and *ν* are the parameters of the algorithm, defining contribution of each term into *C*_1_(*p*). The meanings of all terms are listed below.

1. The first term in the expression of *C*_1_ shows how close the generated probabilities are to the ones assigned by the experts.
2. The second term accounts for the graph geometry and guarantees that the objects close to one another on the graph will have similar probability distributions over classes.
3. The third term is included in case other terms in the expression are not contradictory. Its purpose is to produce a regular and uniform probability distribution over classes.

Numerically, the problem is solved using Alternating Minimization (AM) [13]. Note that *D*_*u*_ is absent in the case of unlabeled data. The minimization of the objective min⁡_*p*_ *C*_1_(*p*) leads to the set of probabilities *p*_*i*_ = {*p*_*i*_^1^,…, *p*_*i*_^*m*^} for each document *d*_*i*_ ∈ *D*. We introduce a threshold *T* ∈ [0,1] to assign additional categories relevant to each document if *p*_*i*_^*j*^⩾*T* then *d*_*i*_ ∈ cl_*j*_.

### 3.2. Weighted kNN Algorithm for Finding Missing Labels

In this section, we shall briefly describe the weighted k-nearest neighbour algorithm [17] that is capable of directly solving the multilabel categorization problem.

Let *ρ*(*d*, *d*′) be a distance function between the documents *d* and *d*′. The function which assigns document *d* to class label cl⁡∈*L* is then defined as

(6) $$\begin{array}{c} S\left(d,\mathrm{cl}\right)=\frac{\sum_{d^{\prime }\in \text{kNN}(d)}\rho \left(d,d^{\prime }\right)I\left(d^{\prime },\mathrm{cl}\right)}{\sum_{d^{\prime }\in \text{kNN}(d)}\rho \left(d,d^{\prime }\right)},\\ I\left(d^{\prime },\mathrm{cl}\right)=\{\begin{array}{cc} 1, & \text{if}\;d^{\prime }\in \mathrm{cl}\\ 0 & \text{otherwise}. \end{array} \end{array}$$

Here, kNN(*d*) is the set of k nearest neighbours of document *d* in the training set.

We introduce a threshold *T* such that if *S*(*d*, cl⁡)⩾*T* then *d* ∈ cl⁡. Then, the algorithm counts *S*(*d*, cl⁡) for all possible (*d*, cl⁡) combinations. When *S*(*d*, cl⁡)⩾*T*, every combination is considered to be a missing label and used to modify the training set.

### 3.3. Support Vector Machine

We will use the Linear Support Vector Machine as a classification algorithm in this case. Since SVM is mainly a binary classifier, the Binary Relevance approach is therefore chosen to address multilabel problems. This method implies training a separate decision rule *w*_*l*_ *x* + *b*_*l*_ > 0 for every category *l* ∈ *L*. More details are available in our previous work on methods for structuring scientific knowledge [18]. In our study, as the implementation of SVM we used Weka binding of the LIBLINEAR library [19].

### 3.4. Random Forest

Random Forest is an ensemble of machine learning methods which combines tree predictors. In this combination, each tree depends on the values of a random vector sampled independently. All trees in the forest have the same distribution. More details about this method can be found in [20, 21]. In our study, we used the implementation of Random Forest from Weka [22].

## 4. Experimental Results

In this section, we describe how did we perform text classification experiments. We applied the classification algorithms to initial (unmodified) training sets as well as to the training sets modified with “add” or “del” methods.

We shall first discuss the scheme of training set transformation and its usefulness. Then, we shall present the process of data generation. Finally, we shall consider the performance measures used in the experiments, experimental setting, and the results of the parameter estimation and final validation.

### 4.1. Training Set Modification

Training set modification step is described in detail in Section 3 (methods). However, it is important to notice that, in both cases, documents that do not belong to set PC according to the relevance algorithm *ψ* (too far from documents labeled to the given category) are still retained in the training set. The reason for this choice is that we assume that experts are not supposed to make any mistake of type II (when they give a document an odd label): only the absence of proper label is supposed to encounter.

The omission of the relevance pair (*d*, *A*) in the training set makes document *d* move into the set of negative examples for learning a classifier for the class *A*. This problem alters the decision rule and negatively affects performance. The proposed set modification scheme is designed to avoid such problems during the training session of the classifier.

### 4.2. Datasets and Data Preprocessing

#### 4.2.1. AgingPortfolio Dataset

The first experiment was carried out using data from the AgingPortfolio information resource [18, 23]. The AgingPortfolio system includes a database of projects related to aging and is funded by the National Institutes of Health (NIH) and the European Commission (EC CORDIS). This database currently contains more than one million projects. Each of its records written in English, displays information related to the author's name, title, a brief description of the motivation, and research objectives, the name of the organization, and the funding period of the project. Some projects contain additional keywords with an average description in 100 words. In this experiment, we used only the title, a brief description, and tag fields.

A taxonomy contains 335 categories with 6 hierarchical levels used for document classification. A detailed information about the taxonomy is available on the International aging research portfolio Web site [23]. Biomedical experts manually put the category labels on the document training and test sets. In the process, they used a special procedure for labeling the document test set. Two sets of categories, carefully selected by different experts, were assigned to each document of the test set. Then, a combination of these categories was used to achieve a more complete category labeling. Different participants like the AgingPortfolio resource users, created the training set with little control. A visual inspection suggests that the training set contained a significant number of projects with incomplete sets of category labels. The same conclusion is also achieved by comparing the average number of categories per project. This average is 4.4 in the sample set compared to 9.79 in the more thoroughly designed test set. The total number of projects was 3 246 for the training set, 183 for the development set, and 1 000 for the test sets.

Throughout our study, we used the vector model of text representation. The list of keywords and their combinations from the TerMine [24] system (National Centre for Text Mining or NaCTeM) provided the terms used in our study. The method used in this system combines linguistic and statistical information of candidate terms.

Later, we conducted the analysis and processing of the set of keyword combinations. Whenever the short keyword combinations were present in longer ones, the latter were split into shorter ones. The algorithms and the code used for the keyword combinations decomposition are available from the AgingPortfolio Web site [8]. According to the results of our previous experiments, the new vectorization method provided a 3% increase by the *F*_1_-measure compared to the general “bag-of-words” model. We assigned feature weights according to the TFIDF rule in the BM25 formulation [25, 26] and then normalized vectors representing the documents in the Euclidean metric of *n*-dimensional space.

#### 4.2.2. Yeast Dataset

Yeast dataset [27] is a biomedical dataset of Yeast genes divided into 14 different functional classes. Each instance in the dataset is a gene, represented by a vector whose features are the microarray expression levels under various conditions. We used it to reveal, if our methods are suitable for the classification of genetic information as well as for textual data.

Let us describe the methods for an incomplete dataset modeling. Since the dataset is well annotated and widely used, the objects (genes) have complete sets of category labels. By random deletion of labels from documents, we made a model of the incomplete sets of labels in the training set. Parameter *p* was introduced as the fraction of deleted labels.

We deleted labels using the following conditions:

1. for each class, *p* represents the fraction of deleted labels. We keep the distribution of labels by categories after modeling the incomplete sets of labels;
2. the number of objects in the training set remains the same. At least one label is preserved after the label deletion process.

No preprocessing step was necessary because the data is supplied already prepared as a matrix of numbers [27].

### 4.3. Performance Measurements

The following characteristics were used to evaluate and compare different classification algorithms:

- Microaveraged precision, recall, and *F*_1_-measure [27];
- CROC curves and their AUC values computed for selected categories [28]. CROC curve is a modification of a ROC curve, where *x* axis is rescaled as *x*_new_(*x*). We used a standard exponent scaling *x*_new_(*x*) = (1 − *e* ^−*αx*^)/(1 − *e* ^−*α*^) with *α* = 7.

### 4.4. Experimental Setup

The procedures for selecting important parameters of the algorithms outlined are described next.

#### 4.4.1. Parameters for SVM****

*AgingPortfolio Dataset. *The following SVM parameters were tuned for each decision rule:

- cost parameter *C* controls a trade-off between maximization of the separation margin and minimization of the total error [15];
- parameter *b*_*l*_ that plays the role of a classification threshold in the decision rule.

We performed a parameter tuning by using a sliding control method with 5-fold cross-validation according to the following strategy. The *C*-parameter was varied on a grid, followed by *b*_*l*_-parameter (for every value of *C*) tuning for every category. A set PC = {(*C*, *b*_*l*_)}_*l*∈*L*_ of parameter pairs was considered optimal if it maximized the *F*_1_-measure with averaging over documents. While *C* has the same value for all categories, the *b*_*l*_ threshold parameter was tuned (for a given value of *C*) for each class label *l* ∈ *L*.

*Yeast Dataset. *In experiments with the Yeast dataset, the selection of SVM parameters was not performed (i.e., *C* = 1, *b*_*l*_ = 0 for all values of *l* ∈ *L*).

#### 4.4.2. Parameters for RF

The 30 solution trees were used to build the Random Forest. The number of inputs to consider while splitting a tree node is the square root of features' number. The procedure was done according to the [29] Leo Breiman, who developed the Random Forest Algorithm.

#### 4.4.3. AgingPortfolio Dataset

Parameters *k* and *T* were tuned on a grid as follows. We prepared a validation set *D*_dev_ of 183 documents as in the case of the test set. The performance metrics for SVM classifiers trained on modified document sets were then evaluated on *D*_dev_. A combination of parameters was considered optimal if it maximized the *F*_1_-measure. Parameter *μ* of the SoftSL algorithm was tuned on a grid keeping *k* fixed at its optimal value. A fixed category assignment threshold of *T* = 0.005 is used for the SoftSL training set modification algorithm. We used *ν* = 0, since all documents in the experiments contained some category labels and regularization was unnecessary.

#### 4.4.4. Yeast Dataset

The method for selecting the parameters for the Yeast dataset is the same as in the AgingPortfolio. *D*_dev_ was composed of 300 (20% of 1 500) randomly selected genes for training. The SoftSL training set modification algorithm was not used for this dataset.

### 4.5. A Comparison of Methods

#### 4.5.1. AgingPortfolio

We evaluated the general performance based on a total of 62 categories that contained at least 30 documents in the training set and at least 7 documents in the test set. The results for precision, recall and *F*_1_-measure are presented in Table 1. It is evident that a parameter tuning significantly boosts both precision and recall.

Also, all of our training set modification methods pay lower precision for higher recall values. If we consider *F*_1_ measure as a general quality function, such trade-off may look quiet reasonable, especially for add+WkNN method.

The average numbers of training set categories per document and documents per category are listed in Table 2. As we can see, the SoftSL approach alters the training set more significantly. As a result, a larger number of relevancy tags are added. This is consistent with higher recall and lower precision values of add+SoftSL and del+SoftSL as compared to WkNN-based methods in Table 1.

**Figure 2:**
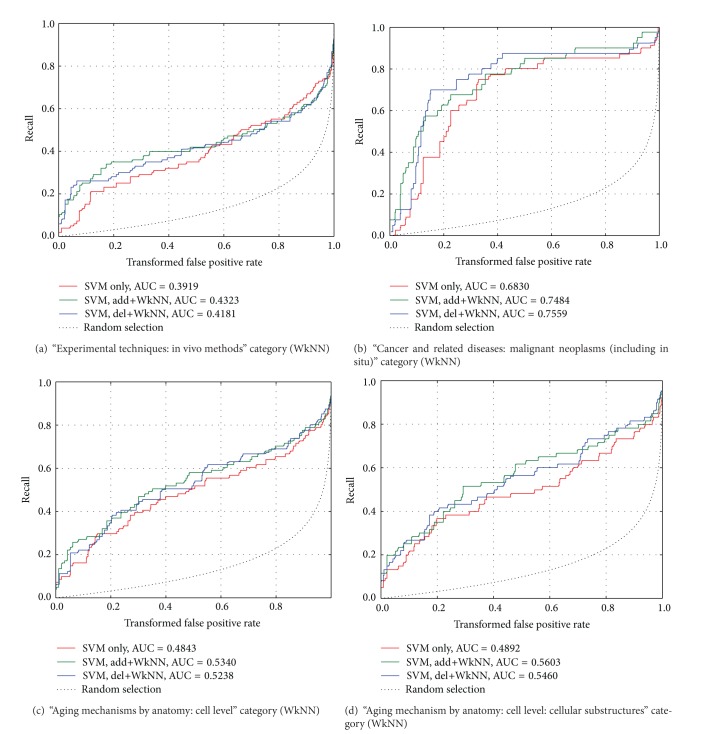
CROC curves for different categories of AgingPortfoliio (SVM classification with WkNN analysis).

Figure 2 compares CROC curves for representative categories of AgingPortfolio dataset computed for SVM without training set modifications, SVM with del+WkNN modification, and SVM with add+WkNN modification. We can notice that SVM classification with incorporate training set modification outperforms simple SVM classification.

**Figure 3:**
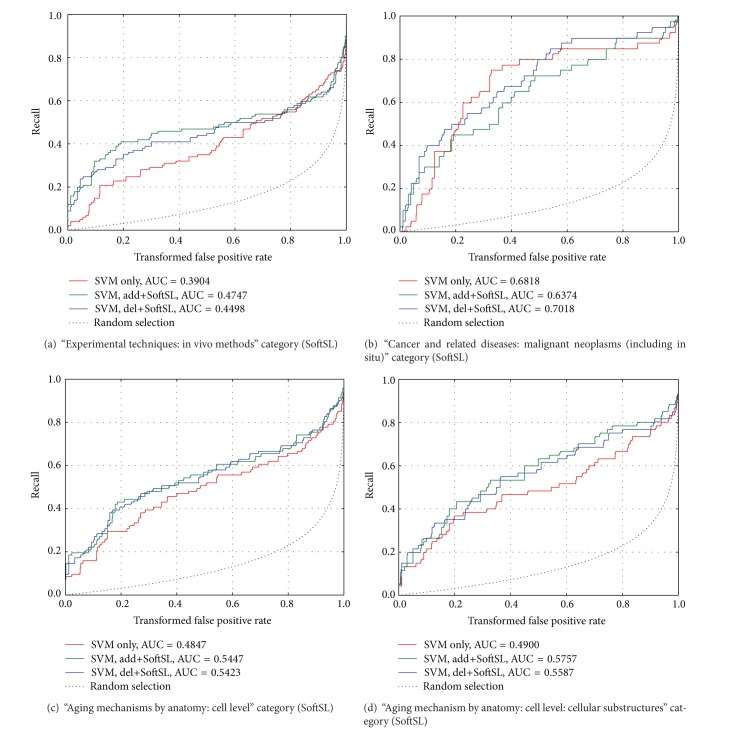
CROC curves for different categories of AgingPortfoliio (SVM classification with SoftSL analysis).

AUC values calculated for the del+WkNN curves are generally only slightly lower, and in some cases even exceed the corresponding values for add+WkNN. A similar situation can be seen in Figure 3 where CROC curves are compared with SVM, del+SoftSL and add+SoftSL.

**Figure 4:**
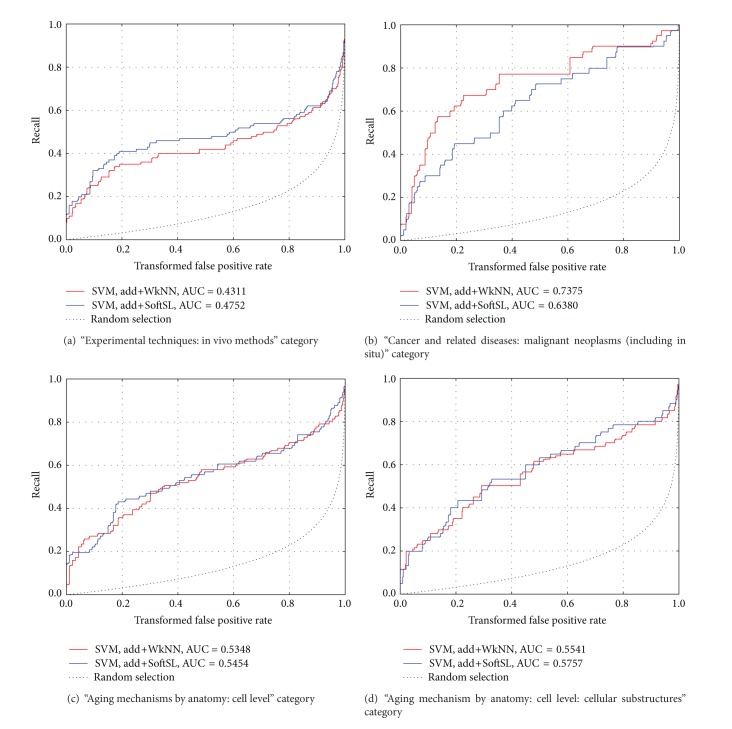
Comparison of WkNN and SoftSL analysis with SVM classification: CROC curves for different categories of AgingPortfolio.

CROC curves for add+WkNN and add+SoftSL SVM classifiers are compared in Figure 4. It is difficult to determine a “winner here.” In most of the cases, the results are pretty equivalent. Sometimes add+WkNN looks slightly worse than add+SoftSL and sometimes add+WkNN has a good advantage against add+SoftSL.

Additional data relevant to the algorithm comparison is presented in Tables 3, 4, 5, and 6. There are precision, recall and *F*_1_ measure for different categories taken with different methods. These results are more relief: it can be seen that add+WkNN outperforms the other methods.

Some values of the metrics of the Random Forest Classification experiments are provided in Table 7. The results in Table 7 show that the modification of the training sets improves the classification performance in this case as well.

#### 4.5.2. Yeast Dataset: The Comparison of the Experimental Results

The dataset is made of 2 417 examples. Each object is related to one or more of the 14 labels (1st FunCat level) with 4.2 labels per example in average. The standard method [30] is used to separate the objects into training and test sets so that the first kind of sets contains 1500 examples and the second contains 917.

The method for modeling the incomplete dataset and the comparison is described above in Section 4.2.2. We created 6 different training sets by deleting a varying fraction (*p*) of document-class pairs. The concrete document-class pairs for deletion were selected randomly.

We proceeded some classification experiments before and after modifying the training set. To compare the methods, we also included the classification results obtained by the SVM or RF on the original nonmodified training set with the complete set of labels (*p* = 0). Here, *p* ∈ {0.1,0.2,0.3,0.4,0.5,0.6} is used.

The results of SVM classification with add+WkNN training set modification, presented in Table 9, show that this modification significantly improves the *F*_1_ measure in comparison with raw SVM results (Table 8).

*A Notable Fact.* Add+WkNN slightly reduced the precision on low *p*, but in the worst cases, with *p* = 0.3 and *p* = 0.4 the precision even rose up. However, the significant improve of recall in all cases is a good trade-off. Recall also significantly improved when the RF algorithm was used in addition to this method (Tables 10 and 11).

## 5. Discussion

Our experiments have shown that the direct application of SVM or RF gives unsatisfactory results for incompletely labeled datasets (i.e., when for each document in our training set some correct labels may be omitted). The case of incompletely labeled dataset strikingly differs from the PU-learning (learning with only positive and unlabeled data) http://citeseerx.ist.psu.edu/viewdoc/summary?doi=10.1.1.91.9914, http://dl.acm.org/citation.cfm?id=1401920 approach: in case of PU training some of dataset items are considered fully labeled, and the other items are not labeled at all.

To overcome the problem, we proposed two different procedures for training set modification, WkNN and SoftSL. Both of these approaches are intended to restore the missing document labels using different similarity measurements between each given document and other documents with similar labels.

We trained both SVM and RF on several incompletely labeled datasets with pretraining label restoration, and without it. According to our experimental results, the label restoration methods were able to improve the performance of both SVM and RF. In our opinion, WkNN works better than SoftSL: it has a better *F*_1_-measure than SoftSL, and, at last, it is simpler to implement.

Furthermore, the comparison of CROC curves for the different methods demonstrated that the classifiers perform slightly worse for some categories and better for others. This pattern appears for classifiers trained on document sets where elements, identified as relevant, are removed from the negative examples. These observations can be attributed to better tuning of the classification threshold as additional relevant documents are added. This is a particularly important aspect for categories containing a small number of documents where additional information about a given category allows better selection of the classification threshold.

One more problem is the evaluation of the incompletely labeled dataset classification results and performance, since the labels in the test set are incomplete as well. One way to overcome this problem is to perform the additional manual post factum validation: any document classification result should be reviewed by the experts in order to reveal if it was assigned any odd labels. Otherwise, the observed results are guaranteed to be lower than the real ones.

Another way to evaluate the classification results and performance is to artificially “deplete” a completely-labeled dataset. We did it with the Yeast dataset. Our experiments with the modification methods applied to artificially partially delabeled Yeast biological dataset confirmed that our approach significantly improves classification performance of SVM on incompletely labeled datasets.

Moreover, the experimental results presented in Section 4.5.2 prove a notable aspect. When we artificially made an incompletely labeled training set and then used our label restoration techniques on it, the *F*_1_ measure for SVM classification was even greater, then for the original, completely-labeled set.

Hence, we are confident that combining the WkNN training set modification procedure with the SVM or RF algorithms will be practically useful to scientists and analysts when addressing the problem of incompletely labeled training sets.

## Figures and Tables

**Table 1:** Microaveraged precision, recall, and *F*_1_-measure (*F*_1_), obtained on AgingPortfilio dataset with different classification methods.

Method	Precision	Recall	*F*_1_
SVM with fixed parameters	0.8649	0.1983	0.2977
SVM with parameter tuning	0.7727	0.3302	0.4159
SVM, del+WkNN	0.5538	0.4452	0.4439
SVM, add+WkNN	0.4664	0.5684	0.4707
SVM, del+SoftSL	0.2132	0.6914	0.3259
SVM, add+SoftSL	0.3850	0.5639	0.4576

**Table 2:** Average number of categories per document and documents per category in AgingPortfolio training set before and after modification.

Modification method	Categories per doc.	Docs. in category
No modification	4.4	45.09
Add+WkNN	15.15	155.14
Add+SoftSL	16.6	168.87

**Table 3:** Microaveraged results for category “experimental techniques: in vivo methods” (AgingPortfilio dataset).

Method	Precision	Recall	*F*_1_
SVM only	0.6	0.12	0.2
With del+WkNN	0.7879	0.26	0.391
With add+WkNN	0.66	0.33	0.44
With del+SoftSL	0.2140	0.64	0.3208
With add+SoftSL	0.4653	0.47	0.4677

**Table 4:** Microaveraged results for category “cancer and related diseases: malignant neoplasms including in situ” (AgingPortfilio dataset).

Method	Precision	Recall	*F*_1_
SVM only	0.4444	0.4	0.4211
with del+WkNN	0.1277	0.9	0.2236
with add+WkNN	0.24	0.9	0.3789
with del+SoftSL	0.0549	1.0	0.1040
with add+SoftSL	0.1032	0.975	0.1866

**Table 5:** Microaveraged results for category “aging mechanisms by anatomy: cell level” (AgingPortfilio dataset).

Method	Precision	Recall	*F*_1_
SVM only	0.5167	0.3827	0.4397
With del+WkNN	0.5946	0.2716	0.3729
With add+WkNN	0.4123	0.5802	0.4821
With del+SoftSL	0.5342	0.4815	0.5065
With add+SoftSL	0.4182	0.5679	0.4817

**Table 6:** Microaveraged results for category “aging mechanisms by anatomy: cell level: cellular substructures” (AgingPortfilio dataset).

Method	Precision	Recall	*F*_1_
SVM only	0.52	0.2167	0.3059
With del+WkNN	1.0	0.0167	0.0328
With add+WkNN	0.4493	0.5167	0.4806
With del+SoftSL	0.75	0.15	0.25
With add+SoftSL	0.3667	0.55	0.44

**Table 7:** Microaveraged results for AgingPortfolio dataset obtained with Random Forest Classification with different training set modifications.

Method	Precision	Recall	*F*_1_
RF only	0.4738	0.2033	0.2467
With del+WkNN	0.4852	0.2507	0.2870
With add+WkNN	0.3058	0.4255	0.3194

**Table 8:** Microaveraged results for SVM, trained on “incompletely labeled” Yeast dataset (with different fraction of deleted labels *p*).

*p* parameter	Precision	Recall	*F*_1_
0	0.7176	0.5707	0.6358
0.1	0.7337	0.5233	0.6109
0.2	0.7354	0.4056	0.5229
0.3	0.6260	0.2442	0.3513
0.4	0.3544	0.1191	0.1783
0.5	0	0	0
0.6	0	0	0

**Table 9:** Microaveraged results for SVM, trained on “incompletely labeled” Yeast dataset (with different fraction of deleted labels *p*) with add+WkNN label restoration. Optimal WkNN parameters *k* and *T* are acquired via grid search.

*p* parameter	Optimal *k*	Optimal *T*	Precision	Recall	*F*_1_
0	—	—	—	—	—
0.1	10	0.3	0.6582	0.6847	0.6712
0.2	10	0.25	0.6525	0.6811	0.6665
0.3	10	0.15	0.6357	0.7137	0.6725
0.4	10	0.1	0.6604	0.669	0.6648
0.5	10	0.05	0.6225	0.7259	0.6702
0.6	10	0.05	0.6248	0.7261	0.6716

**Table 10:** Microaveraged results for RF, trained on “incompletely labeled” Yeast dataset (with different fraction of deleted labels *p*).

*p* parameter	Precision	Recall	*F*_1_
0	0.6340	0.5087	0.5315
0.1	0.6081	0.4613	0.4959
0.2	0.5693	0.3648	0.4133
0.3	0.5788	0.3240	0.3873
0.4	0.5068	0.2471	0.3094
0.5	0.5194	0.2431	0.3104
0.6	0.4354	0.1621	0.2224

**Table 11:** Microaveraged results for RF, trained on “incompletely labeled” Yeast dataset (with different fraction of deleted labels *p*) with add+WkNN label restoration. Optimal WkNN parameters *k* and *T* are acquired via grid search.

*p* parameter	Optimal *k*	Optimal *T*	Precision	Recall	*F*_1_
0	—	—	—	—	—
0.1	10	0.3	0.5940	0.7120	0.6224
0.2	10	0.25	0.5626	0.7462	0.6146
0.3	10	0.15	0.5282	0.7992	0.6095
0.4	10	0.10	0.5223	0.7648	0.5940
0.5	10	0.05	0.4672	0.8388	0.5726
0.6	15	0.05	0.4547	0.8695	0.5707

## References

[1] Hosseini SM, Abdi MJ, Rezghi M (2012). A novel weighted support vector machine based on particle swarm optimization for gene selection and tumor classification. *Computational and Mathematical Methods in Medicine*.

[2] Li SC, Liu J, Luo X (2013). Iterative reweighted noninteger norm regularizing svm for gene expression data classification. *Computational and Mathematical Methods in Medicine*.

[3] Gao Z, Su Y, Liu A, Hao T, Yang Z (2013). Nonnegative mixed-norm convex optimization for mitotic cell detection in phase contrast microscopy. *Computational and Mathematical Methods in Medicine*.

[4] IKatakis I, Tsoumakas G, Vlahavas I (2010). *Mining Multi-Label Data, in Data Mining and Knowledge Discovery Handbook*.

[5] Forman G (2003). An extensive empirical study of feature selection metrics for text classification. *Journal of Machine Learning Research*.

[6] Yizhang G (2010). *Methods for Pattern Classification*.

[7] Leopold E, Kindermann J (2002). Text categorization with support vector machines. How to represent texts in input space?. *Machine Learning*.

[8] International Aging Research Portfolio http://www.agingportfolio.org/wiki/doku.php?id=datapage.

[9] Esuli A, Sebastiani F Training data cleaning for text classification.

[10] Tang J, Chen Z, Fu AW, Cheung DW (2007). Capabilities of outlier detection schemes in large datasets, framework and methodologies. *Knowledge and Information Systems*.

[11] Zagoruiko NG, Borisova IA, Dyubanov VV, Kutnenko OA (2008). Methods of recognition based on the function of rival similarity. *Pattern Recognition and Image Analysis*.

[12] Lee LH, Wan CH, Yong TF, Kok HM (2010). A review of nearest neighbor-support vector machines hybrid classification models. *Journal of Applied Sciences*.

[13] Subramanya A, Bilmes J Soft-supervised learning for text classification.

[14] Subramanya A, Bilmes J Entropic graph regularization in non-parametric semi-supervised classification.

[15] Joachims T Text categorization with support vector machines: learning with many relevant features.

[16] Hart P, Cover T (1967). Nearest neighbor pattern classification. *IEEE Transactions on Information Theory*.

[17] Raghavan P, Manning CD, Schuetze H (2009). *An Introduction to Information Retrieval*.

[18] Zhavoronkov A, Cantor CR (2011). Methods for structuring scientific knowledge from many areas related to aging research. *PLoS ONE*.

[19] Fan R-E, Chang K-W, Hsieh C-J, Wang X-R, Lin C-J (2008). LIBLINEAR: a library for large linear classification. *Journal of Machine Learning Research*.

[20] Breiman L (2001). Random forests. *Machine Learning*.

[21] Díaz-Uriarte R, Alvarez de Andrés S (2006). Gene selection and classification of microarray data using random forest. *BMC Bioinformatics*.

[22] Hall M, Frank E, Holmes G, fahringer BP, Reutemann P, Witten IH (2009). The weka data mining software: an update. *ACM SIGKDD Explorations Newsletter*.

[23] http://agingportfolio.org.

[24] Frantzi K, Ananiadou S, Mima H (2000). Automatic recognition of multiword terms. *International Journal of Digital Libraries*.

[25] Robertson SE, Walker S, Jones S Okapi at trec-3.

[26] Sebastiani F (2005). Text categorization. *Text Mining and Its Applications*.

[27] Godbole S, Sarawagi S Discriminative methods for multi-labeled classification.

[28] Swamidass SJ, Azencott C-A, Daily K, Baldi P (2010). A CROC stronger than ROC: measuring, visualizing and optimizing early retrieval. *Bioinformatics*.

[29] http://www.stat.berkeley.edu/~breiman/RandomForests/cc_manual.htm.

[30] Elisseeff A, Weston J (2001). *Advances in Neural Information Processing Systems*.

